# “It is worth hanging in there” – Psychotherapeutic experiences shaping future motivation for outpatient psychotherapy with refugee clients in Germany

**DOI:** 10.1186/s12888-023-05004-3

**Published:** 2023-07-12

**Authors:** Flurina Potter, Marlene Zehb, Katalin Dohrmann, Veronika Müller-Bamouh, Brigitte Rockstroh, Anselm Crombach

**Affiliations:** 1 Department of Psychology, University of Konstanz, Konstanz, Germany; 2 vivo international, Konstanz, Germany; 3 Department of Psychology, Saarland University, Saarbrücken, Germany

**Keywords:** Refugees, Mental health, Psychotherapy, Therapists, Qualitative research, Content analysis, Challenges, Health care, Germany

## Abstract

**Background:**

A high prevalence of mental disorders in refugees contrasts with a low rate of treatment and limited access to health care services. In addition to pre-, peri- and post-migration stress, language, cultural barriers together with lack of information about cost reimbursement, and access to German (mental) health care institutions are discussed as barriers to use of available services. Such barriers together with insufficient experience of treating traumatized refugee clients may lower therapists’ motivation and facilities to accept refugee clients. A model project called “Fearless” trained, and supervised therapists, translators, and peer counsellors to reduce these barriers and increase therapists’ motivation and engagement in future treatment of refugees.

**Methods:**

From a total 14 therapists participating in the project *N* = 13 were available for semi-structured interviews. The interviews were scheduled during or after their outpatient psychotherapy of refugee clients and lasted one hour on average. Based on qualitative assessment strategies, open questions addressed the therapists’ experience of challenges, enrichments, and motivation throughout the therapy. Therapists’ responses were analyzed using content structuring qualitative content analysis.

**Results:**

Three major challenges modulated therapists’ future motivation for treating refugee clients: specific bureaucratic efforts (e.g., therapy application), organizational difficulties (e.g., scheduling appointments), and clients’ motivation (e.g., adherence, reliability). Still, most interviewed therapists (*n* = 12) evaluated the therapy as enriching and expressed their motivation to accept refugee clients in the future (*n* = 10).

**Conclusion:**

Results recommend the reduction of bureaucratic effort (e.g., regular health insurance cover for all refugees) and implementation of organizational support (e.g., peer counsellors) in support of therapists’ motivation for future treatment of refugee clients. Further structural support e.g., with organizing and financing professional translators and referring refugee clients to psychotherapists should be deployed nationwide. We recommend the training in, and supervision of, the treatment of refugee clients as helpful additional modules in psychotherapy training curricula to raise therapists’ motivation to work with refugee clients.

**Supplementary Information:**

The online version contains supplementary material available at 10.1186/s12888-023-05004-3.

## Background

In 2021, 49.4% of asylum applicants in Germany were younger than 18 years [1]. Another 15.8% were between 18 and 25 years old [2]. Their integration into their host nation society remains a challenge not only for Germany, but for many countries [3, 4]. A high prevalence of illness, in particular of mental disorders, has been verified, potentially resulting from substantial pre-, peri-, and post-migration stress [3–5]. A recent meta-analysis estimated prevalence rates as high as 29.9% for post-traumatic stress disorder (PTSD) and 39.8% for depression amongst newly arrived refugees and asylum seekers in Germany [6, 7]. Similar prevalence rates were found in adolescent refugees in Europe: about half were affected by PTSD and up to one-third by emotional or behavioral problems, such as depression or anxiety disorders [8].

Despite the obvious need for treatment, the number of refugees admitted to adequate treatment in Germany was rather low [9–12]. A recent nationwide survey of the health care situation of refugees confirmed that mental health care was not sufficiently ensured for refugees [13]. Moreover, according to psychosocial care reports in Germany, referrals of refugee clients to psychotherapists in private practice became more difficult and decreased in recent years [14, 15]. Several barriers impair the access to adequate mental health care services. While a physical examination is required by law upon arrival in Germany, a mental health screening has not been implemented despite European guidelines and research recommendations [9–11, 16, 17]. In addition to language challenges, refugees reported missing information about mental health services [18] and the structure of the German health care system [11, 19]. During the first 18-months, most asylum seekers in Germany do not have regular health insurance. Hence only treatment for pain and acute illnesses, vaccinations, emergency and maternity care is covered [10, 14, 20]. Additional services -such as psychotherapy- must be approved by municipalities on a case-by-case basis [10].

Reports indicated further challenges for therapists providing psychotherapy for refugees: organizational challenges, such as increased waiting times for therapy funding approval of refugee clients without regular health insurance, insufficient collaboration of the different mental health services and, lack of financial compensation for psychotherapists for additional work, missed appointments and translators [21, 22]. Treatment-related challenges include insufficient knowledge in dealing with (traumatized) refugees, treatment of clients from other cultures, and insufficient availability of trained translators [21–23]. Post-migratory stressors such as uncertain residency status, limited work permits, and placement in refugee shelters may be topics of therapy sessions rather than mental health issues, thereby complicating the therapy process further [21, 24]. Lack of experience and expertise in treatment of refugees may result in a fear of contact [14, 15]. In a vignette experiment with German licensed psychotherapists, Dumke and Neuner [25] illustrated this fear of contact: when comparing psychotherapists’ attitudes towards outpatient treatment of refugee clients and non-refugee clients with the same symptoms, attitudes towards refugee clients were found to be less favorable. The psychotherapists expected more difficulties and negative emotions in treatment of refugee clients, which fostered the tendency to refuse an outpatient treatment of refugee clients. Furthermore, correlations between less refugee clients treated in the last twelve months; less training attended on the topic as well as more therapy-hindering attitudes were found. In contrast, previous therapeutic experience with this client group was associated with an increased willingness to accept refugee clients [26].

Against this background of refugee’s high need for mental health services but limited health care access and low admission to psychotherapy, the model project “Fearless” was developed in Baden-Württemberg. The project aimed at facilitating adolescent refugees access to mental health services. The project was funded by the Foundation Baden-Württemberg in cooperation with the Competence Center for Psychotraumatology of the University of Konstanz, as well as the Lake Constance Institute for Psychotherapy (apb) and the NGO “vivo international.“ The project aimed at implementing an established training plan for the screening and treatment of adolescent refugees within German healthcare structures. Refugee clients were recruited by social workers at the refugee shelters, who informed potential participants, mostly between the ages of 14 and 22, about the project. They were screened with the Refugee Health Screener [27–29] by trained psychologists with a doctoral, master’s or bachelor’s degree, who worked for the “Fearless” project. Psychologically distressed refugees were offered psychotherapy within the project, or a referral to other services. Translators and peer counsellors were trained in translation in psychosocial contexts, mediating between cultures and navigating the German health care system. In addition, peer counsellors were instructed on how to support clients during their access to health care services and treatment with respect to organizational or psychological aspects (e.g., accompanying them to- and reminding them of- individual appointments). Therapists received four days of training in the diagnosis and treatment of post-traumatic disorders using Narrative Exposure Therapy (NET) and Forensic Offender Rehabilitation NET (FORNET), an adaptation of NET for offenders with a low aggression threshold [30, 31]. Thereafter, therapists were supervised by supervisors who received project supervision in NET and FORNET if needed.

Aiming to understand and overcome barriers to healthcare for refugee clients, we focused on the perception of challenges as well as the motivation of German therapists providing psychotherapy to said clients. As the main providers of psychotherapy, the readiness of psychotherapists to engage with refugee clients is essential to tackle refugees’ high prevalence of mental health disorders and their adverse effects on integration.

## Methods

### Participants

Table 1 provides an overview of the demographic information of the 13 interviewed therapists. Most of the therapists were female (*n* = 11), still in the process of becoming licensed psychotherapists^1^, and two therapists were working as licensed psychotherapists in private practice.

**Table 1:** Demographic Data of the Interviewed Therapists

Category	Count (in %)	Category	Count (in %)
Gender		Age	
Female	11 (85)	25–29	3 (23)
Male	2 (15)	30–39 40–49 50–59	5 (38) 4 (31) 1 (8)
Outpatient therapy sessions^1^			
100–250		3 (23)	
300–450		2 (15)	
> 600		5 (38)	
No information		3	

***Notes***. ^1^ As part of the German psychotherapy training therapists monitor the amount of conducted outpatient therapy sessions of 50 min.

The interview responses were based on the therapists’ treatment of 22 refugee clients, 15 to 41 years old. Table 2 provides the demographic information of these clients, their most frequent diagnoses as well as further clinical information. Most of the clients were male, and more than 50% had no regular health insurance. The most prevalent diagnosis was PTSD.

**Table 2:** Demographic Data of Refugee Clients

Category	Count (in %)	Category	Count (in %)
Gender		Age	
Female	2 (9)	Mean	22.22 years
Male	20 (91)	SD	6.13 years
Regular Health Insurance		Peer Counsellors	
With Without	10 (45.5) 12 (54.5)	With Without	9 (41) 13 (59)
Therapy Status		Most Frequent Diagnoses^1^	
No Successful Referral Ongoing Therapies Regular End of Therapy Dropout Before 6th Session Dropout After 6th Session	4 (17) 5 (22) 6 (26) 2 (9) 5 (22)	PTSD Only PTSD + Depressive Disorder PTSD + Substance dependency No Diagnosis	6 (27) 8 (36) 2 (9) 4 (18)
Most Frequent Countries of Origin^2^			
Afghanistan	4 (18)		
Guinea	4 (18)		
Syria	4 (18)		
Gambia	3 (14)		

***Notes***. ^1^ After successful referral the treating therapists gave the diagnoses. Other diagnoses included: PTSD + psychotic disorder, other childhood emotional disorders (one each). ^2^ Other countries of origin: Palestine, Senegal, Somalia, Ethiopia, Eritrea, Iraq, Iran (one each).

#### Data collection

Therapist interviews addressed their personal experience with the treatment of refugee clients from the Fearless project. Qualitative data were collected using a semi-structured interview guide. In line with suggestions of Helfferich [33] the interview guide consisted of four open questions about organizational and content implementation, culture as an influencing factor, and the therapists’ personal experience. Potential factors influencing these four themes were rephrased into questions and grouped under the narrative-inspiring questions.

The purposive sampling was an attempted full survey: all 14 external therapists who received at least one referral from a refugee client in the Fearless project were invited to participate in one interview. Participants were invited to participate in the study via mail by the project manager and informed about the content of the study: experiences and specifics of psychotherapy with refugee clients. Participation was voluntary and therapists received 50 € for participating. Data collection was completed after 13 interviews, when all 14 potential respondents had been asked to participate. One therapist did not respond to the request to participate. In qualitative research, it is assumed that content saturation occurs after twelve interviews [32].

The interviews were conducted by a postdoc and doctoral student who were involved in the project and, hence, knew the therapists. A third interviewer was a master’s student who did not know the therapists. The three women, aged 24, 27, and 37, were Caucasian and spoke German as their native language. The interviewers were introduced into the manual of Helfferich [33] and attended a methods workshop on qualitative research methods. The interview guide was piloted with the first author, who has also worked therapeutically with refugee clients herself. Individual influences were reflected through training on how to conduct interviews and the interview guide. Interviews were conducted from March to May 2022. Due to the Covid-19 pandemic, three interviews were conducted in person at the offices and ten were conducted online via the BigBlueButton video platform. Four interviews were interrupted by technical problems of less than five minutes and continued by telephone. Aiming to ensure the best manual adherence and common understanding amongst the interviewers, six interviews were conducted with another interviewer present. Interviews lasted from 42 to 69 min (*M* = 56 min). All interviews were conducted in German and recorded on a voice recorder. Quotes in support of results were translated back and forth from German to English and from English to German in the research team. All respondents gave their consent for participation, recording of the interview and further processing, securing and anonymization of their interview data. Prior to the interview, respondents completed a brief demographics questionnaire. An interviewer protocol, completed after the interview, noted special incidents. The interviews were conducted as part of the Fearless project and the project was approved by the Ethics Committee of the University of Konstanz.

### Qualitative analysis

The interviews with the therapists were analyzed based on subjective epistemology, thus it was assumed that the interviews provide access to the subjective view of the therapists [34]. An exploratory as well as descriptive and evaluative research design was chosen. Most of the audio recordings were transcribed by the interviewer who conducted the interview. All material was transcribed using standard orthographic transcription, and spoken texts were anonymized and corrected according to current spelling and dialect expressions [35]. After the initial transcription, the transcripts were compared to the audio recording by the transcriber herself or by another interviewer. For reasons of transparency, participants were given the opportunity to view their transcribed interviews. The data was analyzed using the program MAXQDA Plus 2022. The analysis of the interview data of the therapists was carried out by the first author in collaboration with the second author. All material was analyzed using content structuring qualitative content analysis according to Kuckartz and Rädiker [36]. The data was summarized and structured using a category system with main and subcategories. For the analysis, the main codes were constructed deductively and modified inductively after the data analysis began, as suggested by Kuckartz [36]. The interview material was analyzed based on these four questions:

1. Which challenges affect the future motivation of therapists to work with refugee clients?
2. Which measures of the Fearless project were found to be helpful in the implementation of therapy?
3. Which strategies of the therapists were found to be useful in dealing with the challenges?
4. How is the future motivation of therapists to work with refugee clients?

#### Quality criteria

As quality criteria common for quantitative data are not directly transferable to qualitative research [37], criteria in qualitative research reflect internal and external study quality. Internal study quality includes characteristics such as reliability, regularity, intersubjective comprehensibility and, credibility [36]. External study quality, refers to the transferability and generalizability of results [36]. Intersubjectivity, which is achieved by discussing interpretations with others [38], was ensured by regular collegial exchange in peer supervision with the research team. Free segmentation coding as used for the analysis of qualitative data does not allow a random-adjusted coefficient such as the kappa coefficient [36]. In order to improve the code system by more precise code definitions, differently coded text passages were compared by “consensual coding” [39] and discussed. The quality criterion of transparency was also emphasized in research [38], this was achieved by documenting the data collection and analysis processes. For this, the COREQ checklist was completed [40, 41], which includes elements that should be documented in qualitative research reports, see Supplement A. External study quality can further be supported by “peer debriefing” [36]. The research questions, procedure, and results were discussed with other qualitative researchers from the fields of psychology, medicine, and sociology in two methodological workshops at the Ludwig-Maximilians-University of Munich.

## Results

### Qualitative interview main categories

The content structuring qualitative content analysis [36] revealed four main categories: challenges (272 coded text segments), helpful project factors (50), useful strategies for dealing with challenges (96), and personal experience of therapists (157). Table 3 provides a table of categories with sub-codes.

**Table 3:** Table of Categories

	Sub-code
Challenges	Bureaucratic effort of therapy
	Therapy organization
	Therapy motivation
	Other challenges
	No explicit main challenge
Helpful project factors	Diagnostic questionnaires
	NET seminars
	Peer counsellor & translators
	Coverage of costs
	Work relationship with Fearless project
	Supervision through Fearless project
	Other
Useful strategies for dealing with challenges	Useful strategies for dealing with bureaucratic effort of therapy
	Useful strategies for dealing with challenges with therapy organization
	Useful strategies for dealing with challenges with therapy motivation
	Useful strategies for dealing with other challenges
Personal experience of therapists	Future motivation
	Enrichments
	Burdens

### Challenges

#### Bureaucratic effort of therapy

Seven therapists considered the additional workload due to administrative tasks not sufficiently financially remunerated as a major challenge in their work with refugee clients (*“daunting”* T04). Examples were the need to write more psychological certificates and expert opinions during the treatment. Moreover, the procedural requests for cost reimbursement and therapy application were less familiar and more time-consuming. Delayed funding approval (*n* = 2), too few hours (*n* = 2) approved for severely stressed clients, and time-consuming contact with the clients themselves, and with other authorities were added as a further burden.

*“Um, that was really tedious and especially the exchange with the health office and the social welfare office [...]. There was so much additional work around the therapy, which was really nerve-racking, exhausting and insanely time-consuming. And that was really much more stressful than the therapy and the therapeutic content itself.“* (T07)

Of the seven therapists who mentioned a higher bureaucratic effort, two had treated refugee clients with regular health insurance, five had treated clients without it. Six therapists considered the bureaucratic effort for refugee clients as similar as for regular outpatients. Of these, four had treated refugee clients with, and two had treated clients without regular health insurance. Of the latter two, one therapy ended before the therapist had to apply for psychotherapy funding.

#### Therapy organization

Treatment organization was mainly challenged by the clients’ unreliability when it came to showing up on time (*n* = 4), cancelling at short notice (*n* = 2) or not attending at all (*n* = 7). According to seven therapists, refugee clients were often difficult or impossible to reach by telephone:

*“[…] just these basics of making an appointment, that took me sometimes almost two hours just to make a new appointment and reach him, I found that very, very exhausting and um also a bit frustrating.”* (T01)

In three cases regular termination of treatment was impossible, as the clients could no longer be reached. Poor reachability of clients was attributed to them not having SIM card credit, or no access to WIFI in the refugee shelters. Two therapists mentioned forgotten or lost referral slips as challenging. Three therapists reported difficulties in organizing appointments with the different parties: themselves, the clients, and the translators. Further facets of more difficult organization were incompatibility of own and refugee clients working hours, language problems facilitating misunderstandings, clients’ lack of (daily) structure and organization, avoidance of trauma exposure in therapy, sleep disorders, lack of drive, addiction, frequent moves, lack of knowledge about the German healthcare system, and potential cultural misunderstandings:

*“[...] I just know that in other countries it can be different and there you might come, then you’re told “okay come to the doctor tomorrow” and then you just come to the doctor the next day sometime and then that’s just the way it is.”* (T04)

Three of the interviewed therapists did not evaluate the treatment organization as challenging and described their refugee clients as reliable or even *“[…] more reliable than most of my other clients.”* (T13).

#### Therapy motivation

Seven therapists reported that clients showed ambivalent treatment motivation evident in, e.g., non-compliance to agreements, cancelling sessions without or with vague excuses. One therapist concluded that for the client *“[…] the effort was too much”* (T03).

Two therapists felt unsure of their client’s goals and concerns or doubts with respect to the treatment. Culturally different therapy concepts (*n* = 1) or the priority of other topics, such as a German language course (*n* = 1) were mentioned as possible reasons for ambivalent motivation. Two therapists emphasized clients being very motivated, another therapist noticed a motivation increase throughout the course of therapy.

#### Additional challenges

Additional challenges mentioned included the difficult living conditions of refugee clients, especially the housing situation, a lack of social support, and uncertainty due to the asylum process. In some therapies, these issues were oftentimes at the forefront, making it for example difficult to conduct trauma therapy. Insufficient command of the foreign language impaired understanding and promoted misunderstandings. The assistance of translators was described as a *“detour”* (T06), time-consuming and prompting frequent queries and back translations. In particular at treatment onset, the language barrier impaired rapport building. Insufficient language competence also lengthened diagnostic procedures and required repeated validation. A culturally determined, different understanding of role models of men and women was noticed by seven therapists, regarding the extent to which male clients showed weakness and emotions in therapy and engaged in a therapeutic process with a woman. Four therapists reported challenges with potentially shameful, sensitive, or taboo topics in therapy. For instance, narrower family structures would make it difficult to work through family conflicts: *“[…] one does not talk bad about the parents, one respects the parents.”* (T13). Five therapists considered different understandings of psychotherapy and mental illnesses as challenging, e.g.,

*“[...] garage principle: So, you drive the car into the garage, it is repaired and drives out again on the other side and it’s all right, so basically the understanding of therapy was very medically oriented, you go to the doctor, you get a pill [...]”* (T05).

### Helpful project measures

Ten therapists emphasized the peer supervision with fellow therapists, as well as the group and individual supervision as helpful and supportive. Close collaboration and constant availability of the project management was appreciated (*n* = 8). The collaborative work was reported to have provided a good transfer of information regarding therapy funding applications, networking in trauma-specific and non-specific questions, and the as motivational perceived shared *“heart and soul”* (T07).

Four therapists appreciated the additional training in NET. Furthermore, the diagnostic questionnaires prepared by the project team provided helpful orientation and initial structure for six therapists. The projects’ organization of translators and peer counsellors (*n* = 4) was considered particularly helpful for organizing appointments and administrative tasks. On the financial side, the therapists acknowledged that supervision costs for therapists, as well as refugees’ travel costs, translators, and peer counsellors’ payment, were covered by the project.

### Useful strategies for dealing with challenges

Above all, the exchange with the project management, colleagues or peer counsellors was considered a high priority and perceived as a very helpful strategy to deal with the various challenges. Adjusting one’s own expectations of the (pace of) therapy to the clients’ needs and capacities was generally considered a useful strategy. Furthermore, transparent discussion of difficulties with clients was particularly helpful in dealing with challenges of therapy organization (*n* = 6) and motivation. This attitude also included being *“[…] as free as possible from prejudices or taboos […]”* (T10), being informed about the client’s context and applying psychoeducation. In the case of culturally sensitive topics, it was important to be understanding and at the same time extending invitations to talk about these topics. With additional bureaucratic work and therapy organization, it was helpful to delegate tasks to the peer counsellors. Furthermore, it was considered helpful to deal more flexibly with structural obstacles, such as the referral slip, that needs to be provided every quarter in the German health care system. Understanding of clients’ situations (*n* = 5) and accommodating to refugee clients’ appointment difficulties more than to non-refugee clients was considered helpful, as was sending out reminders about appointments and referral slips. In two cases, agreements on cancellation fees improved the clients’ compliance. Setting boundaries was elementary to enhance treatment motivation for therapy, it was also emphasized as useful to adapt the intensity of one’s own work to the client. Overall, therapists stressed keeping a balance between being understanding of the clients’ situation and one’s own needs. Moreover, repeating questions, summarizing, and avoidance of technical language was considered useful to overcome language difficulties. Since topics other than the mental state or traumatic experiences were often the focus of therapy, one therapist conducted an *“everyday life session”* (T07) in addition to a weekly trauma session.

### Therapists’ motivation to engage in further treatment of refugee clients

Therapists described their experiences with refugee clients as being as much of a burden as an enrichment (*n* = 12). In addition to the challenges described above, examples of burdens were difficult biographies (*n* = 7), with topics such as child abduction, experiences of fleeing their home country, or on-going conflicts in the country of origin:

*“[…] it doesn’t leave me unscathed. It does something to you, but I think that’s part of the job and I can deal with that quite competently.”* (T07).

For three therapists, the feeling of one’s own incompetence, of inexperience and of insecurity was burdensome. One therapist, who felt that the therapy was not very enriching, cited difficulties to form a relationship with the client because of psychotic symptoms as the reason. Enrichment was seen in learning about an “*unusual story*” (T03), a different upbringing, a foreign country, a different culture, religion, or other perspectives. Eight therapists found the experiences with NET and working with refugee clients enriching for their professional skills. Seven therapists mentioned seeing an improvement in their clients as encouraging.

“*It is worth hanging in there, also in terms of content. You can see a change over the course of treatment. And that has, that was [...] I think not only for the client, but also for me very relieving and motivating to continue.”* (T10)

Regarding their motivation to continue refugee treatment in the future, ten therapists expressed high motivation, two of them felt ready to accept refugee clients again at any time. Three therapists would rather wait until completion of their psychotherapist training. In the context of the psychotherapist training, the time pressure to complete therapies as quickly as possible, and the burden of simultaneously visiting seminars and working at a clinic was too overwhelming. Three therapists could imagine working on a *“small scale”* (T11) with one or two refugee clients in an outpatient setting. Furthermore, a *“better asylum policy in Germany”* (T12), regular access to health insurance for all refugees, and practical support for refugees, e.g., for doctor visits or reminders about therapy, would increase therapist’s motivation. Likewise, a faster processing time for therapy funding and closer cooperation with social workers in the refugee shelters would be essential.

## Discussion

Challenges occurring during outpatient psychotherapy with refugee clients affecting therapists’ motivation for future treatment were examined in interviews with therapists during or after their outpatient psychotherapy of said clients. Moreover, factors of the Fearless project perceived as helpful and therapists’ useful strategies in dealing with these challenges were examined. Therapists noted three main challenges influencing their future motivation for the treatment of refugees: bureaucratic effort of therapy, therapy organization, and clients’ therapy motivation.

Especially the bureaucratic effort of therapy with refugee clients has been addressed in previous research in Germany [21, 22]. In this research, mainly therapists who had treated refugee clients without health insurance found the bureaucratic effort challenging. In consequence, it is possible that the challenging additional bureaucratic effort mostly depended on the health insurance situation of the refugee clients. German asylum politics might further increase the work burden by requesting expert opinions from psychotherapists [22] which involve additional paperwork. Handing out health insurance cards on a regular basis to all refugees at their arrival has already been proposed in research [21], and could be an important first step in lowering bureaucratic obstacles to receiving adequate psychotherapeutic care. The potential of such a solution has been demonstrated in the case of more than over one million Ukrainian refugees entering Germany in 2022 [42, 43] who were given regular health insurance access on arrival. Furthermore, research has shown that instead of saving money, health insurance access restrictions for refugees led to more hospital and emergency admissions and ultimately higher health care expenditures compared to granting regular access [10, 44]. In conclusion, bureaucratic hurdles can be supported as a main reason for a difficult referment of refugee clients to psychotherapists [14, 15].

Many therapists struggled with therapy organization, e.g., with appointment cancellations, poor reachability, and in some cases low reliability of the refugee clients. Past research has mentioned that cancelled appointments placed therapists in financial dilemmas, and poor reachability and relocation of refugee clients were the most frequent reasons cited for therapy dropout [15, 21, 45]. In this study, therapists mostly attributed difficult therapy organization to the refugees’ living situation, such as frequent moves due to residence requirements [46], or to highly prevalent mental health issues [47]. Implementing peer counsellors in every therapy with refugees might reduce these organizational challenges. Lastly, it is worth mentioning that not every therapist encountered difficulties with the therapy organization.

Therapists’ perception of a challenging therapy organization was possibly intertwined with the third challenge, namely refugee clients’ ambivalent therapy motivation. Amongst other reasons, therapists perceived non-compliance to agreements or the cancelling of sessions without or with vague excuses to be due to the clients lacking therapy motivation. Possibly, the unsuccessful referrals and the dropout of several clients might have added to the perception of an ambivalent therapy motivation [14, 15]. The possible lack of motivation and dropout might partially be attributed to the fact that the refugee clients in our study were mainly male adolescents. According to previous research, lower consultation rates and help-seeking patterns were reported in men than in women [48], and adolescent males in particular had the highest dropout rates of mental health services [49].

One therapist in our study referred to the clients’ different therapy concepts as a potential factor influencing the therapy motivation of the refugee client. Indeed, Murphy and Rosen [50] proposed that differing assumptions between clients and therapists about an illness model as well as coping mechanisms might well contribute to an ambivalent therapy motivation or therapists’ perception thereof. Different therapy concepts have also been reported to be challenging in other studies with refugee clients [45, 51], evidenced by different illness models, and resulting in different or unrealistic expectations of clients towards therapy [51]. The challenge of dealing with different therapy concepts might have been attenuated by the following factors of the Fearless project: providing information about psychotherapy very early on, i.e., during the initial screenings, supporting therapies with peer counsellors often sharing a similar cultural background as the refugee clients, and providing ongoing supervision for psychotherapists, peer counsellors and translators, thereby reinforcing a common understanding of psychotherapy. Other factors influencing therapy motivation, e.g., that caution and mistrust on part of the refugee client can sometimes be misunderstood as a lack of motivation for therapy [15], or that ambivalent therapy motivation has also been observed in the treatment of PTSD in other populations [50], were not mentioned by the therapists of our study.

Other challenges described in research were found in this study too: difficulties with gender roles [52], therapists struggling with their clients’ difficult living situations [21], practical issues being at the forefront of the therapy [24] and feeling burdened by hearing refugee clients’ difficult biographies [21, 22, 45]. Some challenges might have been ameliorated by helpful Fearless project factors. Not having contact with the target population [53] and difficulties with organizing and financing professional translators [21, 22, 45, 51], were probably attenuated by the Fearless project organizing these aspects. Furthermore, training, organizing, and implementing peer counsellors might have lessened organizational challenges reported on by therapists. Training for psychotherapists has further been highlighted as important [51, 52] and some challenges, related to high self-doubt, implementation of NET or diagnostic procedures might have been attenuated by training the therapists before beginning the therapy. Lastly, as finding supervisors who have experience working with refugee clients seems to be difficult [52, 54], we conclude that key support structures of the project were the extensive supervision and close collaboration with the project management. This research emphasizes the importance of deploying similar structural support for therapists working with refugee clients nationwide. Several projects [19] in Germany have demonstrated the effectiveness of coordinating the integration of refugee clients in psychotherapy by combining training psychotherapists with the provision of translators and peer counsellors.

Therapists also reported implementing several useful strategies which have already been highlighted in research, such as adjusting methods to the refugee clients [45], psychoeducation [51], appropriate supervision, being open to differences or dealing more flexibly with bureaucratical obstacles [52]. Furthermore, therapists in our study reported transparency, setting boundaries, adapting the intensity of one’s own work to the clients and agreeing on a cancellation fee as useful. Whether these general psychotherapy strategies are of more importance in therapy with refugee clients than with non-refugee clients cannot be deducted from this research. In culture-sensitive psychotraumatology an empathic and non-judgmental attitude and the attempt to understand the client’s individual cultural background is considered of particular importance [55]. Several therapists reported such an attitude as a strategy for dealing with challenges. However, they also stressed the importance of finding a balance between understanding and one’s own boundaries.

Despite encountering several of the above-mentioned challenges, most therapists reported having found the therapy experiences with refugee clients personally and professionally enriching and were motivated to treat refugee clients in the future. This result aligns with research highlighting previous therapeutic experience with refugee clients as a crucial factor for raising therapists’ willingness and lowering therapy-hindering attitudes [25, 26]. Our study underlines the importance of including treatment of refugee clients in psychotherapy training, whilst additional support, including supervision, is still available to the trainees. Even more so, as previous research associated therapy-hindering attitudes with younger age and less work experience [25]. As self-doubt has been associated with a lower readiness to work with refugee clients [26] and lack of expertise can result in a fear of contact [14, 15], we recommend building an expertise with this client group early on.

### Limitations

Several methodological limitations restrict the generalizability of the results, e.g., most interviewed psychologists were in psychotherapy training, and all therapists were supported by the Fearless project. The small number of male participants interviewed reflects the unequal gender distribution of 76% female psychotherapists across Germany [56]. Moreover, in qualitative studies the sample size of 13 interviews is viewed as sufficient for content saturation [32]. One therapist, who had reported poor experiences with several failed transfers of refugee clients could not be included in the study because of not responding to the interview request, which may have distorted the results. Since all interviewed therapists participated in the Fearless project, social desirability could have influenced the answers. We attempted to counter this potential bias by having the second author, who was not part of the project team, interview therapists who knew the other two interviewers. Interview guides and training on how to conduct interviews were used to minimize and reflect potential individual influences.

The interviewers and the majority of interview participants were from western, educated, industrialized, wealthy, and democratic countries [57]. Thöle et al. [21], reported that therapists might attribute statements from refugee clients to cultural differences, even though they might not be culturally valid. The process of a privileged group perceiving a specific subordinate group as deviant from the norm because of internalized stereotypical representations is defined as othering [25, 58]. In inclusionary othering difference may be a “tool for connecting” ([58]; p.26), and can have beneficial consequences for the clients. In this study, therapists reported to have had more understanding for organization difficulties with their refugee clients than with their non-refugee clients. However, an unconscious and uncritical transfer of clichés and stereotypes and a generalization of population differences to single cases should be avoided and can have negative effects on the provision of mental health care for refugees [25]. Ultimately, not all therapists experienced the same challenges with their refugee clients, e.g., one therapist stated her client was even more reliable than other non-refugee clients. Schnyder et al. [55], concluded that it is relevant to be aware of culture-specific aspects, whilst still being beware of premature cultural stereotyping and proposed incorporating people from the client’s same cultural background to the therapy whenever necessary. Lastly, according to subjective epistemology, the described outcomes and identified difficulties do not reflect objective reality, but rather the subjective perceptions of the participants. Perceptions and narratives may be distorted by memory bias, selective perception, social desirability, or other effects.

## Conclusion

Treating refugee clients is important on an individual level but also on a societal level as emotional distress and integration are intertwined [59, 60]. Bureaucratic effort of therapy, therapy organization, and the perceived clients’ therapy motivation crucially influence therapists’ future motivation for the treatment of refugee clients. Our results suggest four main recommendations: Firstly, access to health care should be facilitated by minimizing bureaucratic obstacles, e.g., by providing regular health insurance for all refugees early on. Secondly, we recommend improving living conditions of refugees and deploying peer counsellors in every therapy to facilitate dealing with challenges of therapy organization and therapy motivation. Other challenges described in previous research on mental health care provision of refugee clients might have been alleviated by the Fearless project: organizing and financing professional translators, referring refugee clients, and ensuring supervision. Hence, thirdly, we recommend addressing these aspects on a nationwide structural level to facilitate access to psychotherapy for refugees within the German health care system. Lastly, our fourth recommendation is to include education on the treatment of refugee clients in the curricula of psychotherapy training. Amongst others, this additional training module should provide information that psychotherapy with refugee clients is effective, emphasize techniques which have proven to work universally and/or for these populations, and reinforce capacities of psychotherapists to work with translators and peer counsellors from different cultural backgrounds. Implementing these recommendations is necessary to ensure high motivation of therapists and mental health care of refugees. Ultimately, more psychotherapists might offer therapy concurring with the conclusion of most psychotherapists in this study to continue working with refugee clients in the future despite existing challenges: *“It is worth hanging in there”.*

## Electronic supplementary material

Below is the link to the electronic supplementary material.

Supplementary Material 1: COREQ (COnsolidated criteria for REporting Qualitative research) Checklist

## Data Availability

The interviews used and analyzed during the current study, the interview guide as well as codebook are available from the corresponding author on reasonable request.

## Footnotes

1 To become licensed psychotherapists, psychologists in Germany complete a three-year training after getting their master’s degree.
